# Protective effect of astragalus membranaceus and its bioactive compounds against the intestinal inflammation in *Drosophila*

**DOI:** 10.3389/fphar.2022.1019594

**Published:** 2022-12-12

**Authors:** Jianzheng He, Xu Li, Shipei Yang, Yan Shi, Yuting Dai, Shuzhen Han, Yixuan Wang, Xingyao Lin, Benjun Wei, Yongqi Liu, Minghui Xiu

**Affiliations:** ^1^ Provincial-level Key Laboratory for Molecular Medicine of Major Diseases and The Prevention and Treatment with Traditional Chinese Medicine Research in Gansu Colleges and University, Gansu University of Chinese Medicine, Lanzhou, China; ^2^ College of Basic Medicine, Gansu University of Chinese Medicine, Lanzhou, China; ^3^ Key Laboratory for Transfer of Dunhuang Medicine at the Provincial and Ministerial Level, Gansu University of Chinese Medicine, Lanzhou, China; ^4^ College of Public Health, Gansu University of Chinese Medicine, Lanzhou, China; ^5^ Research Center of Traditional Chinese Medicine in Gansu, Gansu University of Chinese Medicine, Lanzhou, China; ^6^ Clinical College of Chinese Medicine, Gansu University of Chinese Medicine, Lanzhou, China

**Keywords:** inflammatory bowel disease, *Astragalus membranaceus*, intestinal homeostasis, bioactive compounds, *Drosophila melanogaster*

## Abstract

Inflammatory bowel disease (IBD) is characterized by chronic and relapsing intestinal inflammation, which currently lacks safe and effective medicines. *Astragalus membranaceus* (AM), also named Huangqi, is one of the most commonly used fundamental herbs in China. Here, we aimed to investigate mechanism and bioactive compounds of AM on treating sodium dodecyl sulfate (SDS)- induced colitis in *Drosophila* flies. Our data showed that AM extract (AME) supplementation had no toxic effect in flies, and protected flies against SDS-induced lifespan shortening, intestinal morphological damage, and colon length shortening. Moreover, AME supplementation remarkably rescued SDS-induced intestinal stem cell (ISC) overproliferation and increased reactive oxygen species (ROS) level in the intestine. Mechanistically, AME remarkably rescued the altered expression levels of genes and proteins in c-Jun N-terminal kinase (JNK) and JAK-STAT signaling pathways induced by SDS in gut. Additionally, formononetin, isoliquiritigenin, isorhamnetin, astragaloside I, astragaloside III, vanillic acid, and caffeic acid in AM had protection against SDS-induced inflammatory damage in flies. Taken together, AME could ameliorate the intestinal inflammation partially by suppressing oxidative stress-associated JNK signaling and JAK-STAT signaling pathways. AME may provide a theoretical basis for natural medicine toward treating intestinal inflammatory disease in human.

## Introduction

Inflammatory bowel disease (IBD) is a chronic recurrent disease that affects the gastrointestinal tract, which is caused by the interaction of environmental factors, bacterial imbalances, and immune disorders in the genetic background (Graham and Xavier, 2020; Roda et al., 2020). The incidence of IBD is increasing in the world, especially in Asia and Africa (Amcheslavsky et al., 2009). The current therapies for IBD mainly include anti-inflammatory drugs and biologics (Baumgart and Sandborn, 2007). However, some limitations exist in the present therapies for some people, such as drug resistance, unsatisfactory long-term efficacy, and severe systemic side effects. Therefore, it is crucial to devise novel effective and sustained natural products for IBD treatment.

Disruption of intestinal homeostasis leads to the development of IBD. The previous studies have demonstrated that inflammation and infection often lead to intestinal mucosal damage and barrier function impairment (Larabi et al., 2020). Intestinal homeostasis is established and maintained *via* a basal level of intestinal stem cell (ISC) turnover to replace the cells loss (Antonello et al., 2015). Upon inflammation and infection, ISCs undergo rapid proliferation to replenish enough cells in a limited time for regeneration (Amcheslavsky et al., 2009). These processes are regulated by a number of signaling pathways such as JAK-STAT, c-Jun N-terminal kinase (JNK), Wnt and Notch signaling (Biteau et al., 2008; Redhai and Boutros, 2021; Wang et al., 2021). Particularly, JAK-STAT signaling has been demonstrated to be involved in the process of immune response to IBD (Wang et al., 2021). In inflammatory states, JAK-STAT signaling is activated and promotes ISC proliferation and differentiation (Soendergaard et al., 2017; Wang et al., 2021). Oxidative stress has been proved to be involved in the pathogenesis of IBD. After infection in the intestine, reactive oxygen species (ROS) accumulates and activates JNK signaling to promote ISC proliferation (Chen et al., 2020; Nagai et al., 2020). Recently, most studies focus on natural products treat IBD *via* regulating ISC function and epithelial homeostasis.

Currently, the chemical compounds sodium dodecyl sulfate (SDS) and dextran sulfate sodium (DSS) that lead to ISC proliferation and disruption of intestinal integrity are extensively used to induce IBD in many model organisms (Wang et al., 2018; Staats et al., 2019). The fruit fly *Drosophila melanogaster* has become a suitable model for investigating mechanism and treatment strategy of IBD, due to its similar anatomical features with mammal intestine and conserved signaling pathways with mammals (Medina et al., 2022; Yang et al., 2022). Compared to rodent models, fly has many genetic tools, less ethical concerns, low maintenance costs and short generation time, which promotes fly as an ideal model to economical and rapid large-scale screening of therapeuticaly useful natural products. For example, Flos Puerariae extract ameliorated the SDS-induced intestinal inflammation by JAK-STAT signaling, Nrf2/Keap1 signaling in the gut (Yang et al., 2022); Ursolic acid could significantly prevent intestinal injury in SDS-stimulated flies *via* inhibiting ISCs hyperproliferation and JNK/JAK/STAT signaling pathway (Wei et al., 2022).

Natural plants have been used to treat colitis in China for hundred years and have been increasingly recognized worldwide for their low toxicity, low side effects, and well tolerated (Duan et al., 2021; Wang et al., 2021). *Astragalus* membranaceus (AM), also named Huangqi, is one of the most commonly used fundamental foods and herbs in Chinese medicine to treat a wide variety of diseases and body disorders for more than 2000 years. The major components of AM are polysaccharides, flavonoids, and saponins. Calycosin-7-O-β-D-glucoside as one of the flavonids is used as chemical marker in quality analyses of AM(Song et al., 2007). Pharmacological research indicates that the extract component of AM has immunoregulatory, anti-inflammatory, antioxidation, and anti-viral activities (Liu et al., 2017; Shan et al., 2019). Previous studies have shown that administation of AM can alleviate LPS or 2,4-Dinitrobenzene sulfonic acid (DNBS) induced intestinal mucosal damage and promoted tissue repair by inhibiting the expression of inflammatory cytokine in rodents (Ko and Chik, 2009; Cui et al., 2018). AM could attenuate inflammation and oxidative stress in intestinal epithelial cells *via* NF-κB activation and Nrf2 response (Adesso et al., 2018). However, the mechanism of AM on treating IBD and its active ingredients also remains unclear.

In this study, we used the fruit fly *Drosophila melanogaster* as a model to investigate the function and regulatory mechanism of AM extract (AME) on the intestine disruption induced by SDS *in vivo*, and dissect the functional compounds of AME against SDS-induced inflammation. Our results demonstrated that AME supplementation significantly increased survival rates, decreased gut morphological disruption and epithelial cell damage, and restored the activated expression of JAK/STAT signaling and JNK signaling induced by ROS. Key compounds of AM were detected to against SDS-induced inflammatory injury. Together, our studies highlighted a new angle of approaching IBD treatment and further clarified the mechanism of AM treatment for IBD.

## Materials and methods

### *Drosophila* strain and maintenance

The following flies were used: *w* ^*1118*^ (#5905) was obtained from the Bloomington *Drosophila* stock center; esg-Gal4, UAS-GFP was generously provided by Dr. Lihua Jin (Northeast Forestry university, China); gstD1-GFP and 10×STAT92E-GFP lines were kindly gifted from Dr Fengwei Yu (Temasek Life Sciences Laboratory, National University of Singapore, Singapore). Flies were raised on standard medium at 25°C and approximately 65% humidity under a 12 h light/12 h dark cycle as previous report (He et al., 2021). 3–5 day old adult female or male flies were collected using light CO2 anesthesia and allowed to recover for 2 days before further experimentation.

### Drug selection and quantitative analysis

AME was purchased from Shanghai Yuanye Bio-Technology Co., Ltd. (Shanghai, China). AME was diluted in a standard cornmeal-molasses medium to different concentrations (0, 5, 10, and 50 mg/ml). 24 molecules of AME used in this study were categorized into 1) flavonoids, 2) saponins, and 3) others (Table 1). These components were dissolved in ethanol at 20 mM and further diluted to 1 mM in the standard medium. The different concentrations of AME or 1 mM different components were mixed with standard food, the fly eggs were moved in the vial containg fly food with or without AME or components until adult flies hatched. The hatched flies was collected and fed fresh food that is same as growth period food until experiments.

**TABLE 1:** Molecule of AM used in this study.

Classification	Molecule name	Structure	Source
Flavonoids	Quercetin	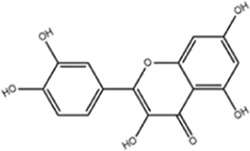	ShanghaiyuanyeBio
	Formononetin	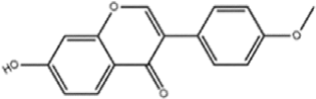	ShanghaiyuanyeBio
	Isoquercitrin	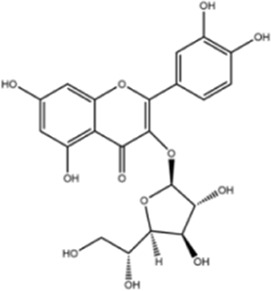	Macklin
	Kaempferol	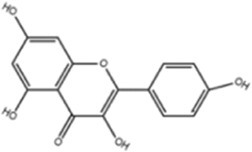	ShanghaiyuanyeBio
	Daidzein	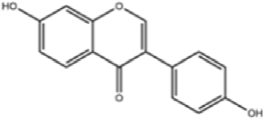	ShanghaiyuanyeBio
	Calycosin	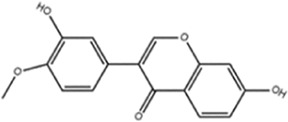	ShanghaiyuanyeBio
	Calycosin-7-O-β-D-glucoside	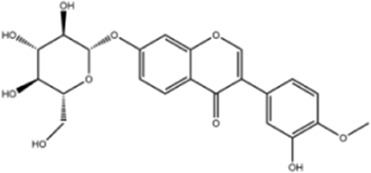	Macklin
	Rutin	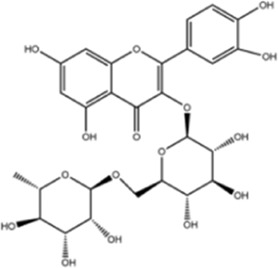	ShanghaiyuanyeBio
	Isorhamnetin	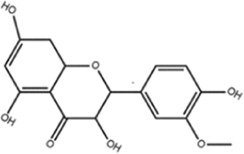	Macklin
	Isoliquiritigenin	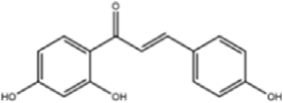	ShanghaiyuanyeBio
	Ononin	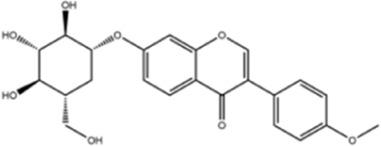	ShanghaiyuanyeBio
Saponins	Betulinic acid	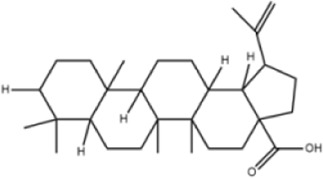	ShanghaiyuanyeBio
	Astragaloside II	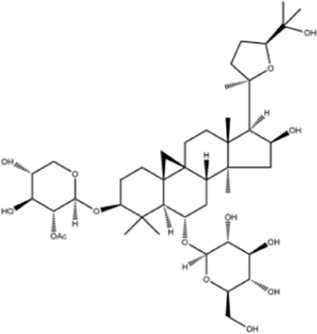	ShanghaiyuanyeBio
	β-asarone	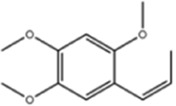	ShanghaiyuanyeBio
	Lupeol	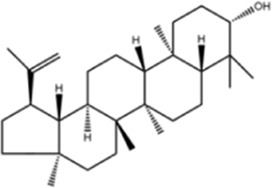	ShanghaiyuanyeBio
	AstragalosideⅠ	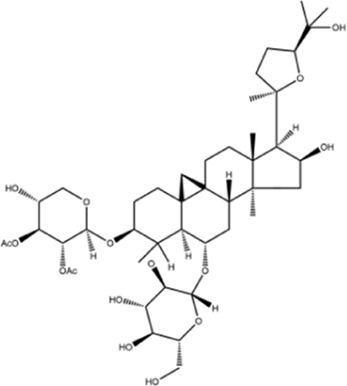	Macklin
	AstragalosideⅢ	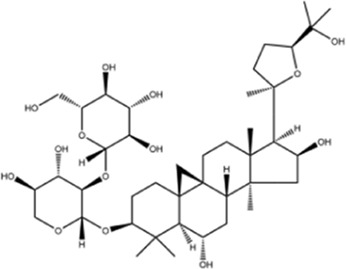	ShanghaiyuanyeBio
	AstragalosideⅣ	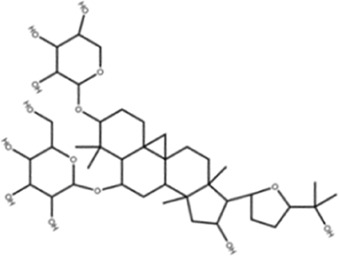	Macklin
Others	Vanillic acid	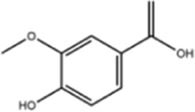	ShanghaiyuanyeBio
	Heriguard	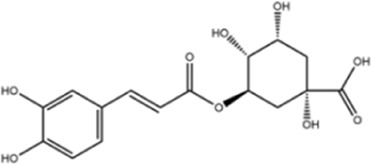	ShanghaiyuanyeBio
	Isoferulic acid	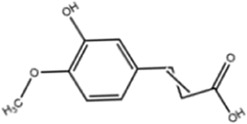	Macklin
	Cis-4-coumaric acid	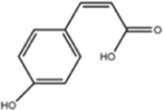	ShanghaiyuanyeBio
	Ferulic acid	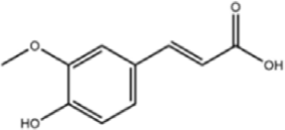	Macklin
	Caffeic acid	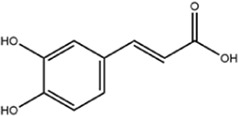	ShanghaiyuanyeBio

Liquid chromatography coupled to mass spectrometry (LC-MS) was used as a reference standard for the quality control of the AME. LC-MS analysis was performed as previously described (Wang et al., 2020). 20 mg AME was mixed thoroughly with 500 μl of aqueous methanol solution (methanol: water 7: 3). The supernatant was collected by centrifugation (12, 000 g) for 5 min at 4°C and filtered with a filter membrane (0.22 μm) before LC-ESI-MS/MS analysis. The sample were analyzed by using an UPLC-ESI-MS/MS system equipped with a Waters ACQUITY UPLC HSS T3 C18 (100 mm × 2.1 mm i.d, 1.8 µm) column maintained at 40°C. Gradient elution of analyses was carried out water with 0.05% formic acid (A) and acetonitrile with 0.05% formic acid (B) at a flow rate of 0.35 ml/min. The gradient elution program was set as follows: 0–1 min, 10%–20% B; 1–9 min, 20%–70% B; 9–12.5 min, 70%–95% B; 12.5–13.5 min, 95% B; 13.5–13.6 min, 95%–10% B; 13.6–15 min, 10% B; After equilibration, 2 μl sample was injected. Data acquisitions were performed using Analyst 1.6.3 software (Sciex). Multiquant 3.0.3 software (Sciex) was used to quantify all metabolites.

### Survival assay

For survival tests under stress conditions, flies fed with or without AME were collected. After starved for 3 h, 20 males or females per vial were transferred into vial containing filter paper soaked in 5% sucrose and 0.6% SDS food with or without AME. The filter papers were changed every 2 days. The dead flies were counted and recorded twice per day. More than 200 flies were scored per group. Three independent experiments were set up.

### Development assay

It was performed as previously described with minor modifications (Yang et al., 2021). Briefly, about sixty eggs per tube were transferred to the vials containing standard food or food with 5, 10, and 50 mg/ml AME, individually. The time that larvae become pupa and the number of pupa in each vial were recorded. After flies hatch, the body weight of adult male or female was measured.

### Blue dye feeding assay

Food intake was performed as previously described (Yang et al., 2021). Briefly, female flies were starved for 18 h, and then were transferred to vials with 5% sucrose and 1% blue dye. Following 6 h of feeding, individual fly was used to measure food consumption under a dissecting microscopy and scoring them according to the relative amount of blue dye in their abdomen. All experiments were conducted blind.

### Intestinal morphology assay

Different groups of female flies were exposed to 5% sucrose with or without 0.6% SDS for 96 h. The gut were dissected in cold PBS and immediately observed under an optical microscope.

### “Smurf” assay

It was performed as previously described with minor modifications (Rera et al., 2011). Briefly, different groups of females were fed in the medium containing 5% sucrose with or without 0.6% SDS for 72 h, then were transferred to vials containing food with a blue dye (2.5% w/v) for 18 h. A fly was remarked as a Smurf when the dye coloration could be observed outside the digestive tract.

### Immunohistochemistry

Female flies supplement with or without AME were exposed to 5% sucrose with or without 0.6% SDS for 16 h, 10–18 females per group were used to dissect intestines in cold PBS, the isolated intestines were fixed with 3.7% formaldehyde for 30 min and washed 3 times with 0.3% PBST. Samples were stained with 4’ 6-diamidino-2-phenylindole (DAPI) for 10 min. The slices were then mounted in Cdear wood oil (Beijing Solarbio Science and Technology Co., Ltd., China) and observed under an Olympus FV1000 Confocal laser scanning microscope (Olympus, Japan). The ISCs proliferation, STAT signaling and gstD1 signaling were identified by the GFP + cell florescence intensity within the posterior midgut. Fluorescence of GFP + cell and their size was measured by using ImageJ.

For pH3 staining, adult female flies were exposed to 5% sucrose with or without 0.6% SDS and incubated at 25 C for 16 h. Intestines were dissected in cold PBS, and fixed with 3.7% formaldehyde for 30 min and washed 3 times with 0.3% PBST. Then the intestines were stained with rabbit pH3 antibodies over night, washed 3 time with 0.3% PBST, and then stained with Alexa 594 anti-rabbit antibody for 3 h and DAPI for 10 min. The slices were then mounted in Cdear wood oil (Beijing Solarbio Science and Technology Co., Ltd., China) and observed under an Olympus FV1000 confocal microscope (Olympus, Japan). The experiments were independently repeated at least three times.

### Reactive oxygen species (ROS) assay

Adult females were fed 5% sucrose medium with or without 0.6% SDS and incubated at 25°C for 72 h. 10–15 intestines per group were dissected in cold PBS and incubated in dihydroethidium (DHE; 30 μM in PBS; Invitrogen) for 5 min in dark environment, then washed 3 times in cold PBST for 10 min, fixed in 3.7% paraformaldehyde for 30 min and immediately observed under a Olympus FV1000 confocal microscope (Olympus, Japan). Single confocal section was used to measure signal intensities using the histogram function in ImageJ. The data presented are from three independent experiments.

### Quantitative RT-PCR analysis

Adult females were fed 5% sucrose with or without 0.6% SDS for 16 h, then total RNA of 60 female guts per group was extracted with TRIzol reagent (Invitrogen) and reverse transcribed into cDNA using Hieff^®^ reverse transcriptase (Shanghai YEASEN, China) according to manufacturer’s instructions. Quantitative PCR was performed with a GFX 96 ConnectTM Optics Module (Bio-Rad Laboratories) using Multiplex PCR Master Mix (Shanghai YEASEN, China). The primer sequences were listed in Table 2. All results were analyzed by the 2-ΔΔCt methods with rp49 as an internal control. The levels of gene expression in all groups were shown as a ratio to the SDS treated group value. At least three replicates were established for each group.

**TABLE 2:** List of forward and reverse used in gene expression study.

Genes	Forward	Reverse
*gstD1*	TGA​TCA​ATC​AGC​GCC​TGT​ACT	GCA​ATG​TCG​GCT​ACG​GTA​AG
*puc*	CGT​CAT​CAT​CAA​CGG​CAA​T	AGG​CGG​GGT​GTG​TTT​CTA​T
*Upd2*	CGG​AAC​ATC​ACG​ATG​AGC​GAA​T	TCG​GCA​GGA​ACT​TGT​ACT​CG
*Upd3*	CCC​AGC​CAA​CGA​TTT​TTA​TG	TGTTACCGCTCCGGCTAC
*Hop*	GTGGGCTCCAAGATACG	GGCAGATACTGAACGGTG
*Socs36E*	CAG​TCA​GCA​ATA​TGT​TGT​CG	ACT​TGC​AGC​ATC​GTC​GCT​TC
*Rp49*	CTTCATCCGCCACCAGTC	GCA​CCA​GGA​ACT​TCT​TGA​ATC

### Virtual screening

The compounds of AM were collected from the traditional Chinese medicine systems pharmacology (TCMSP, https://www.tcmspw.com/tcmsp.php) and Traditional Chinese Integrated Database (TCMID, https://119.3.41.228:8000/tcmid/).

The parameters of absorption, metabolism, distribution, excretion, and toxicity of compounds of AM were calculated by using the ProTox-II data platform. The active compounds were predicted by the QikProp module of Schrödinger.

### Statistical analysis

The data were expressed as the means ± standard error of mean (S.E.M). All experiments were performed with at least three replicates. The significance of statistical differences was analyzed using the GraphPad Prism 8.0. One-way ANOVA test was used to determine statistical significance unless otherwise mentioned. Survivorships among groups were compared and tested for significance with a Log-rank test. The significance level was indicated as *p < 0.05, **p < 0.01, ***p < 0.001.

## Results

### AME increases the survival rate following the ingestion of SDS

**FIGURE 1:**
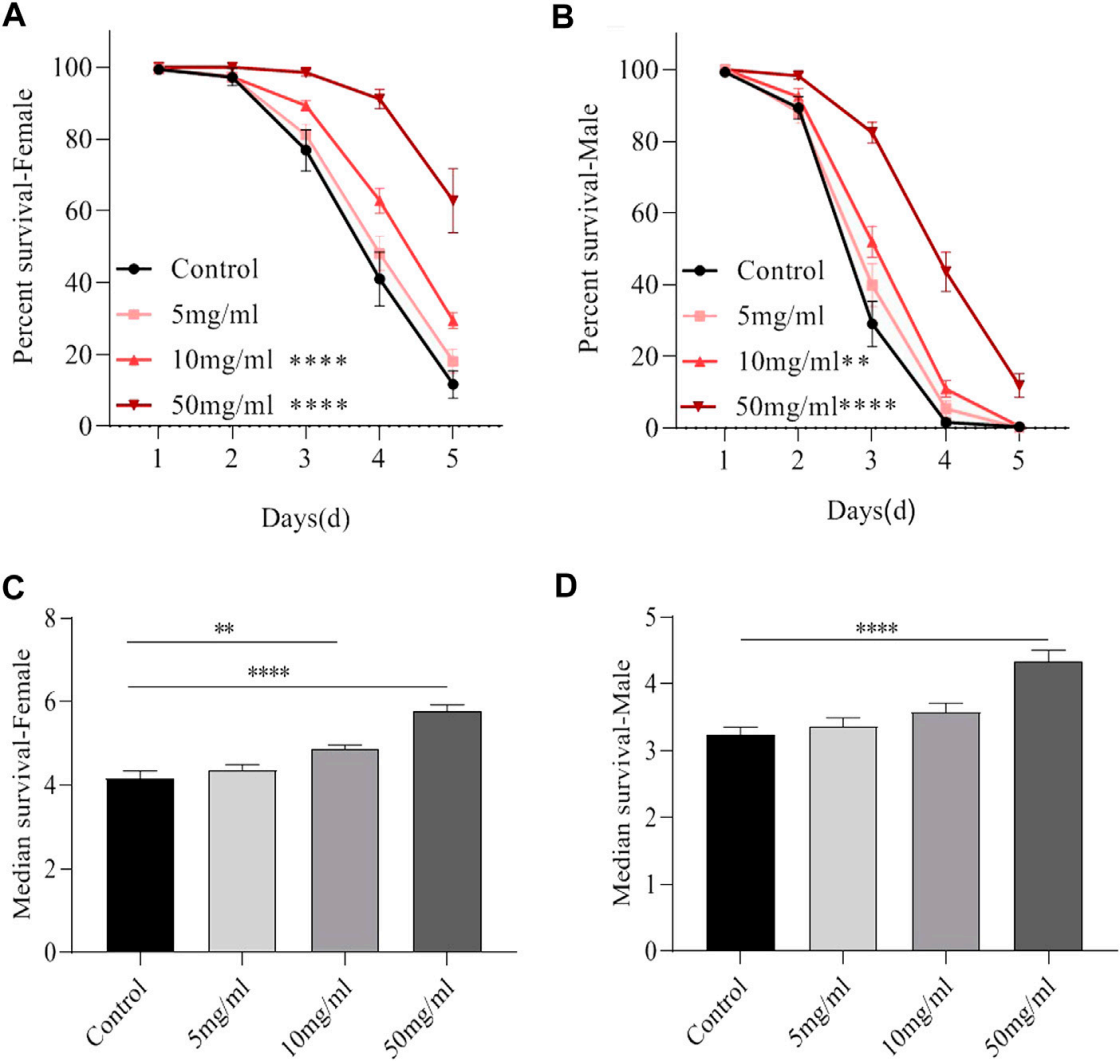
AME improves the lifespan under SDS stimulation. Both 10 mg/ml and 50 mg/ml AME supplementation significantly elevated the decreased lifespan of female **(A)** and male **(B)** flies exposed to 0.6% SDS (*n* = 9–14). The median lifespan in females **(C)** and males **(D)** fed the food with 0.6% SDS. Log-rank *p* values between survival curves are shown. The results are presented as the means ± SEM. **p < 0.01, ****p < 0.0001.

To analyze the anti-inflammatory activities of AME, flies were treated orally with inflammatory reagent SDS. SDS interferes with the normal function of the intestinal barrier and stimulates local and systemic inflammation (Yang et al., 2022). As shown in Figures 1A, B, flies fed with 10 mg/ml and 50 mg/ml AME had extended lifespan under SDS stimulation. The medial survival rates of female and male flies were dramatically increased when flies were fed with AME at 10 and 50 mg/ml, without 5 mg/ml (Figures 1C, D). These results indicated that AME could protect flies against SDS-induced inflammatory injury.

### AME has no toxic effect in flies

**FIGURE 2:**
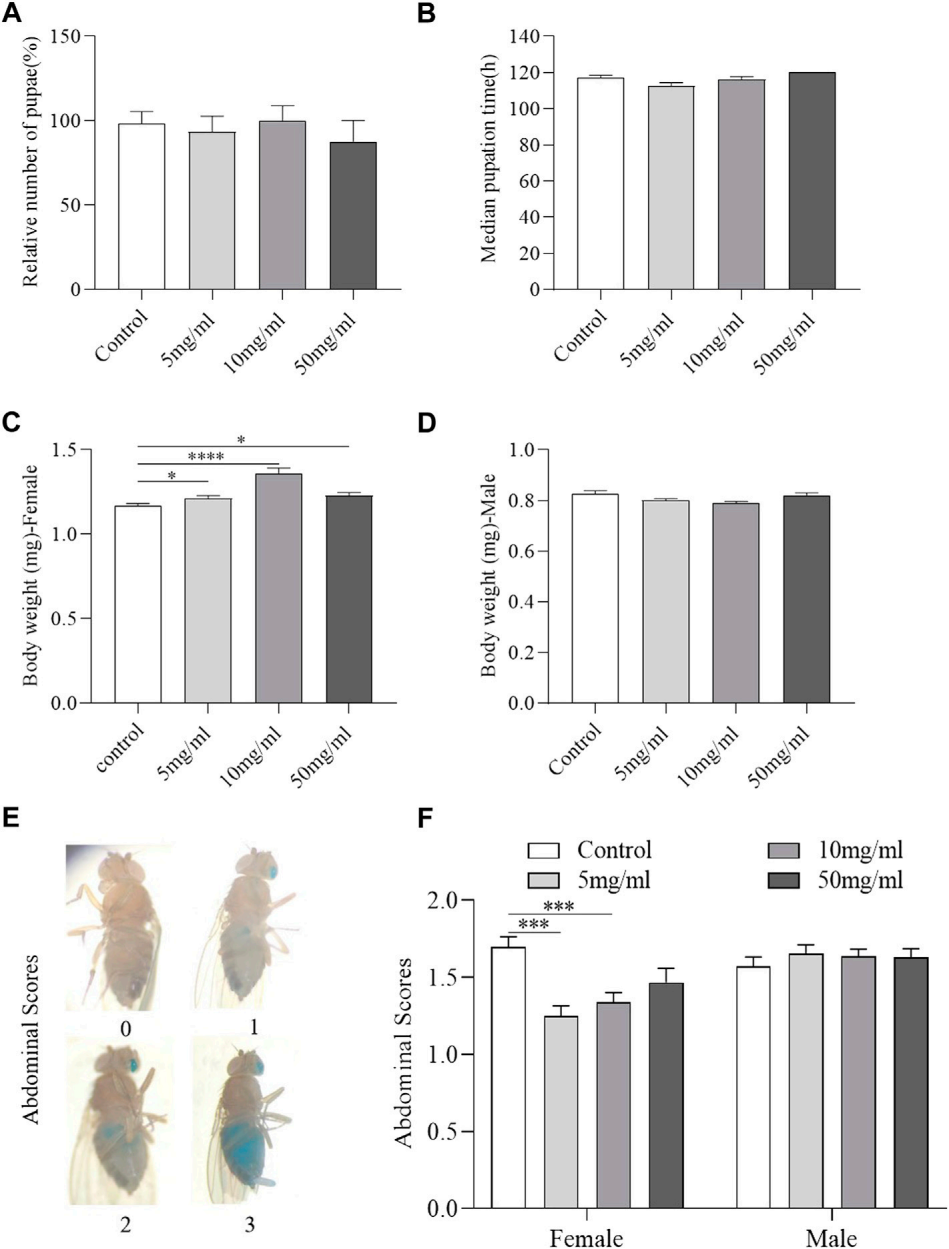
AME has no toxic effect in flies. Flies were develop in standard foods or foods supplemented with AME (5, 10, and 50 mg/L). During their development, AME supplementation did not affect the total number of pupae **(A)** and median time from egg to pupae **(B)** (*n* = 15-16). 5 mg/ml and 10 mg/ml AME supplementation increased body weight of female flies **(C)**, while no significant changes in body weight of male flies **(D)** (*n* = 12–19). Visual scoring analysis of food consumption by utilizing a non-absorbent blue food dye **(E)**. 10 mg/ml and 50 mg/ml AME supplementation reduced the feed intake of female flies (*n* = 60) and had no effect on male flies **(F)** (*n* = 120). The results are presented as the means ± SEM. *p < 0.05, ***p < 0.001, ****p < 0.0001 vs. control group.

Development state of flies is regarded as a high-throughput method for evaluating drug safety (Abnoos et al., 2013). We examined the hatchability and growth rate of flies fed with or without AME. The results showed that flies fed with AME at 5, 10, and 50 mg/ml had similar hatchability and growth rate from 1st instar to pupae stage compared to control flies (Figures 2A, B), indicating that AME had no function to regulate development. Then, the adult body weight and food consumption were determined after flies hatch. Female flies fed with AME at 5, 10, and 50 mg/ml had heavier body weight than control females (Figure 2C), while there was not significantly different body weight between control males and AME-treated males (Figure 2D). Compared to control flies, the total amount of food intake was decreased in females fed with AME at 5 mg/ml and 10 mg/ml, but it was not altered in males fed AME (Figures 2E, F), indicating that AME supplementation mediated metabolism in females. Together, these results indicated that AME had no toxic effect in flies.

### AME alleviates the intestinal morphological damage induced by SDS ingestion

**FIGURE 3:**
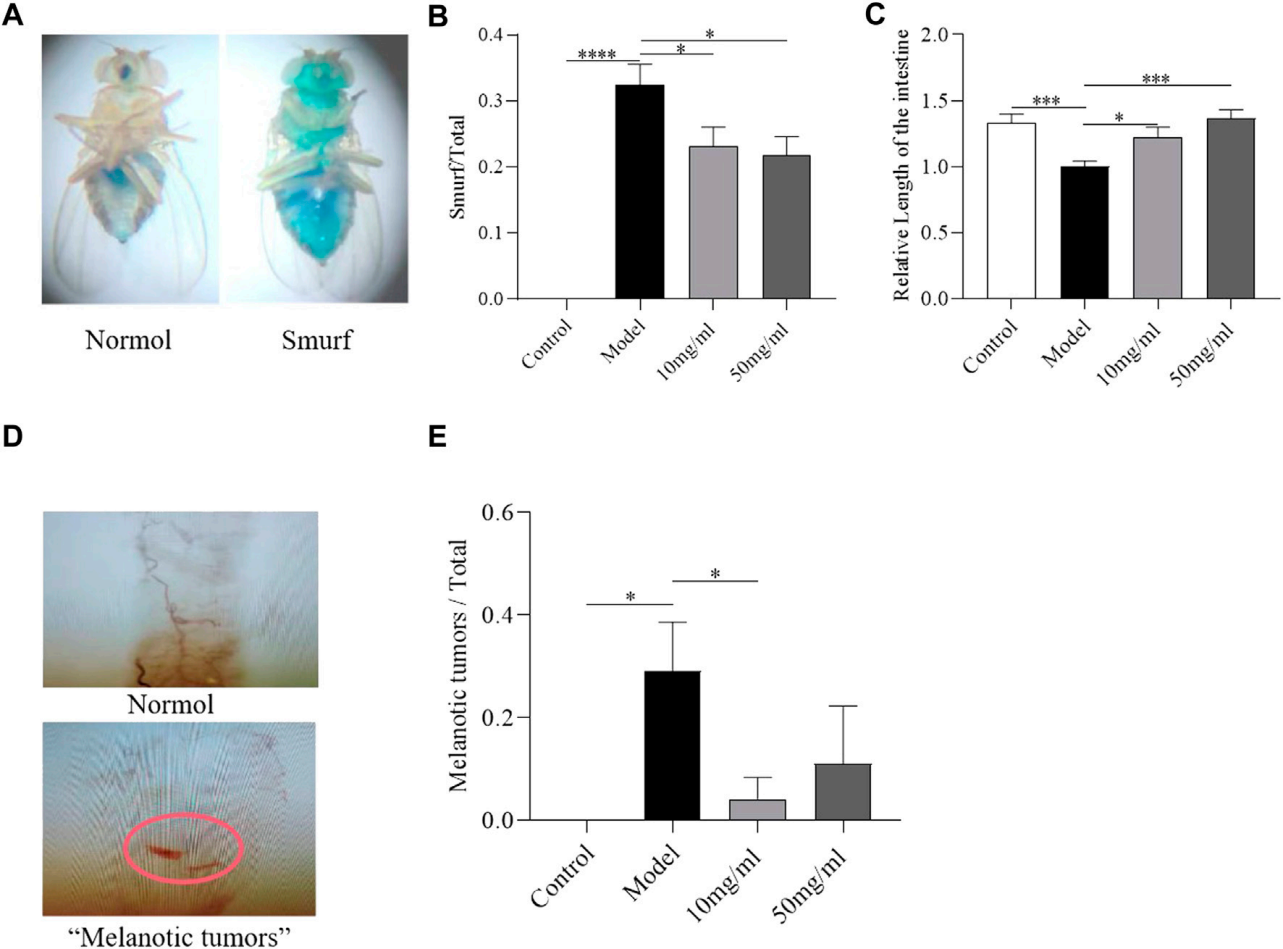
AME ameliorates the SDS-induced intestinal morphological disruption. Gut barrier function was assayed by using “Smurfs” experiments **(A)** Left panel, “Normol”; right panel, “Smurfs”. The percentage of Smurf phenotypes was significantly reduced in flies fed with 50 mg/ml AME **(B)** (*n* = 112–231). 10 mg/ml and 50 mg/ml AME supplementation relieved SDS-induced intestinal shortening **(C)** (*n* = 14). Nomarski images of the fly gut: Up panel, “Normol”; down panel, “Melanotic tumors” **(D)**. 10 mg/ml AME reduced the frequency of melanotic tumors appearing in flies **(E)** (*n* = 9–24). The results are presented as the means ± SEM. *p < 0.05, ***p < 0.001, ****p < 0.0001 vs. control group.

The integrity of intestinal morphology is disrupted in SDS-treated flies (Wei et al., 2022). To investigate the protective effect of AME against morphological disruption in the intestine following SDS stimulation, we determined the intestinal barrier integrity, intestinal length, and “melanotic tumors” in the intestine. The intestinal barrier function was evaluated using the “Smurfs” experiments. Disruption of intestinal barrier leaded to the Smurf phenotype with blue dye throughout the whole body of flies (Figure 3A) (Patel et al., 2019). About 35% SDS-treated flies showed the smurfness (Figure 3B). The percent of “Smurfs” phenotype was significantly decreased in flies fed with 50 mg/ml AME (Figure 3B), indicating that AME alleviated the epithelial damage induced by SDS. Next, we found the intestine length became shorter in SDS-treated female flies compared to control flies (Figure 3C). Both 10 mg/ml and 50 mg/ml AME supplementation remarkably rescued the shortened intestine length in flies under SDS stimulation. In addition, SDS could induce “melanotic tumors” in adult fly intestine (Liu et al., 2016) (Figure 3D). Following treatment with SDS, “melanotic tumors” in the posterior midgut were observed in about 28% SDS-treated flies. 10 mg/ml AME supplementation significantly decreased the frequency of SDS-induced “melanotic tumors”, while 50 mg/ml AME supplementation played a weak function (Figure 3E). Therefore, these results indicated that AME protected against intestinal disruption caused by SDS in flies.

### AME inhibits the stem cell proliferation induced by SDS

**FIGURE 4:**
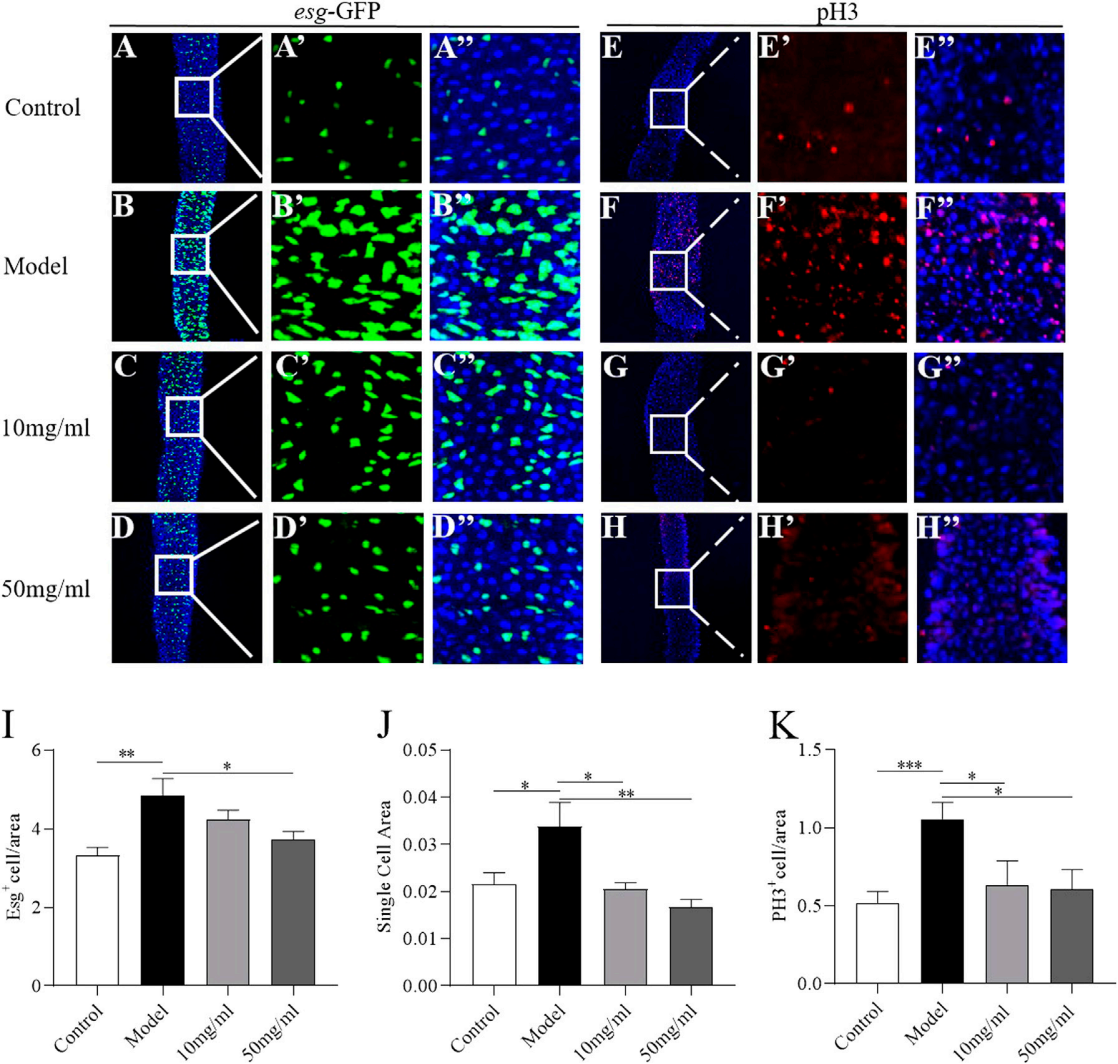
AME protects against SDS-induced ISC proliferation. Immunofluorescence images of the dissected midguts of esg-GFP in adult flies. Esg^+^ cell were labeled with GFP (green) and DAPI (blue). After treatment with SDS for 16 h, Esg^+^ cells were clustered **(A,B″)**. 50 mg/ml AME supplementation significantly rescued the increased GFP targeted cells **(C,D″)**. Quantification of the relative size **(I)** and number **(J)** of Esg^+^ cells (*n* = 15–18). The number of dividing cells was observed by using an anti-pH3 antibody (red). After treatment with SDS for 16 h, PH3^+^ cell were increased **(E, F)**. 10 mg/ml and 50 mg/ml AME supplementation significantly rescued the pH3 marked cells **(G,H)**. Quantification of the number of PH3^+^ targeted cells **(K)** (*n* = 6–11). *p < 0.05, **p < 0.01,***p < 0.001 vs. model group.

Intestinal morphology damage leads to the activation of ISC proliferation to regenerate the damage intestinal epithelium (Buchon et al., 2009). We next evaluated the protective effect of AME on the intestinal homeostasis. Firstly, the transgeneic fly strain, esg-Gal4; UAS-GFP, was used to determine the ISCs and enteroblasts that were GFP positive (Micchelli and Perrimon, 2006). In control flies, just some small and dispersed subset of GFP + cells was found in intestinal epithelium (Figures 4A, A’). After treatment with SDS for 16 h, the GFP + cells were clustered. The number and cell area of GFP + cells significantly increased (Figures 4B, I, J). 50 mg/ml AME supplementation showed a strong reduction in the number of GFP + cells, while 10 mg/ml and 50 mg/ml AME supplementation clearly decreased the cell area of GFP + cells (Figures 4C–J). It suggests that AME can rescue the abnormal proliferation of ISCs and enteroblasts. To further support this finding, we used an anti-phosphohistone H3 (anti-pH3) antibody that marks mitotic stem cells in intestinal epithelium. The number of pH3+ cells increased in fly midguts under SDS stimulation (Figures 4E–F″, K), which is consistent with previous studies (Buchon et al., 2009). 10 mg/ml and 50 mg/ml AME supplementation significantly decreased the increased number of PH3+ cells in SDS-treated flies (Figures 4G–H″, K). Therefore, these results indicated that AME restrained abnormal ISC proliferation induced by SDS in flies.

### JAK-STAT pathway participates in the protection of AME in intestinal homeostasis

**FIGURE 5:**
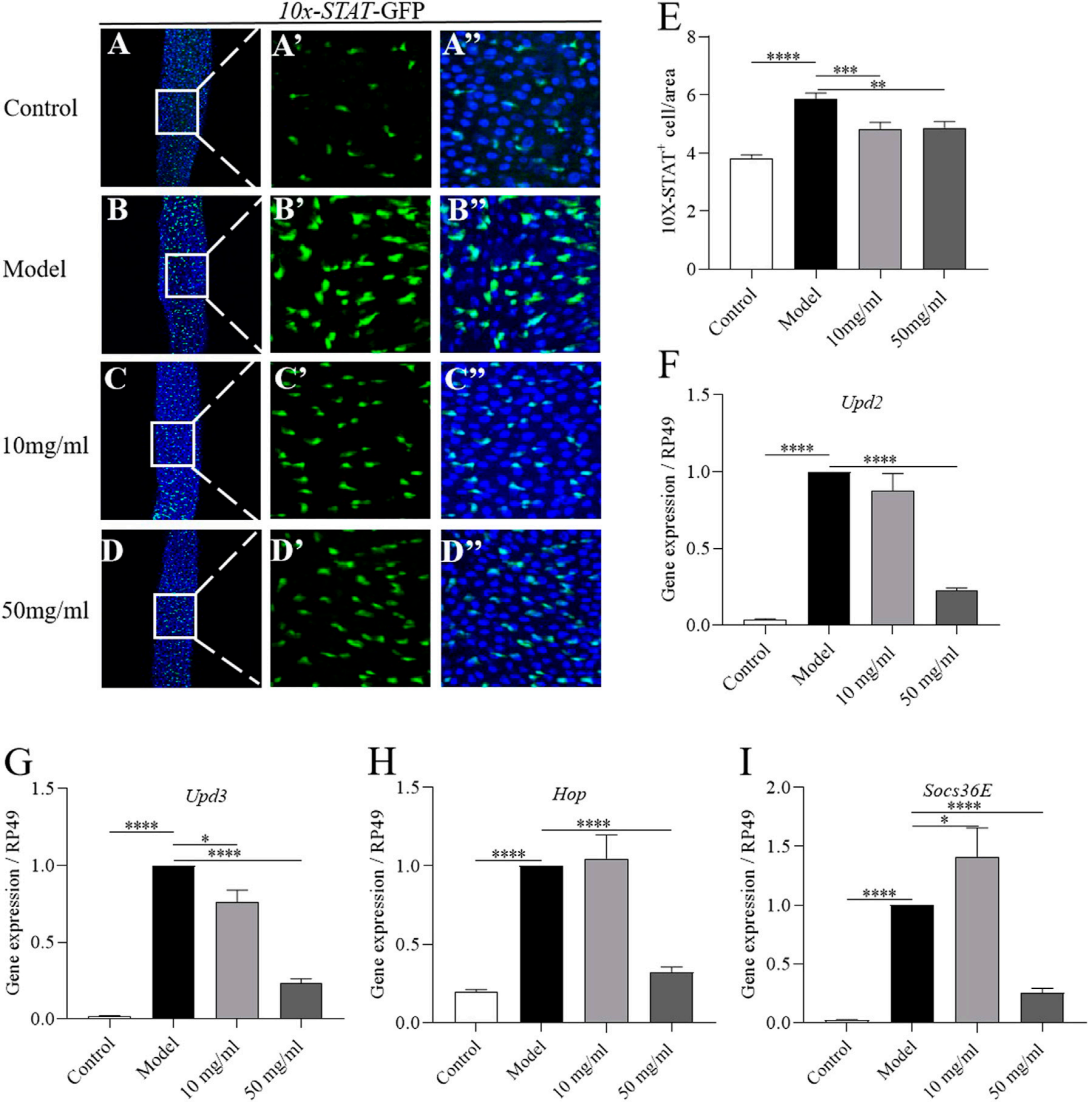
AME prevents SDS-induced ISC proliferation *via* suppressing JAK-STAT pathway. Immunofluorescence images of the dissected midguts of 10×STAT-GFP (green) in flies. After treatment with SDS for 96 h, the level of GFP was remarkably increased in the midgut **(A,B″)**. Supplementation of AME at 50 mg/ml significantly rescued the expression of GFP^+^ cells **(C,D″)**. Quantification of the number of STAT positive cell **(E)** (*n* = 23–37). 10 mg/ml and 50 mg/ml AME supplementation rescued the SDS-induced increased gene expression of *upd2* **(F)**, *upd3* **(G)**, *Hop* **(H)**, and *Socs36e* **(I)** in the gut (*n* = 5-6). The results are presented as the means ± SEM. *p < 0.05, **p < 0.01, ***p < 0.001, ****p < 0.0001.

The JAK-STAT pathway is activated in ISCs by cytokines (Upd2, Upd3) upon SDS stimulation (Buchon et al., 2009). To investigate whether AME protected the SDS-induced intestinal epithelium damage mainly by inhibiting the JAK-STAT pathway, we firstly used a fly line carrying the JAK-STAT pathway reporter gene 10×STAT92E-GFP (Bach et al., 2007). The number of GFP + cells was significantly increased in SDS-treated flies compared to control flies, while 10 mg/ml and 50 mg/ml AME supplementation remarkably rescued the increased level of GFP + cells in SDS-treated flies (Figures 5A–E). To further certificate the protective function of AME depended on JAK-STAT signaling pathway, we examined the expression of genes encoding the components of JAK-STAT pathway-the stimulated cytokines, Upd2 and Upd3; the activated Janus kinase, Hopscotch (Hop); a repressor of the receptor/JAK complex, Socs36E, in the intestine (Jiang et al., 2009). SDS stimulation significantly increased mRNAi levels of Upd2, Upd3, Hop, and Socs36E in the intestine, while 50 mg/ml AME supplementation remarkably rescued the increased expression levels of these genes (Figures 5F–I). 10 mg/ml AME supplementation enhanced Socs36E expression in SDS-treated flies. Collectively, these results suggested that AME had function to protect intestinal homeostasis partly *via* inhibiting JAK-STAT pathway.

### AME alleviates SDS-induced intestine damage by inhibiting oxidative stress-associated JNK signaling

**FIGURE 6:**
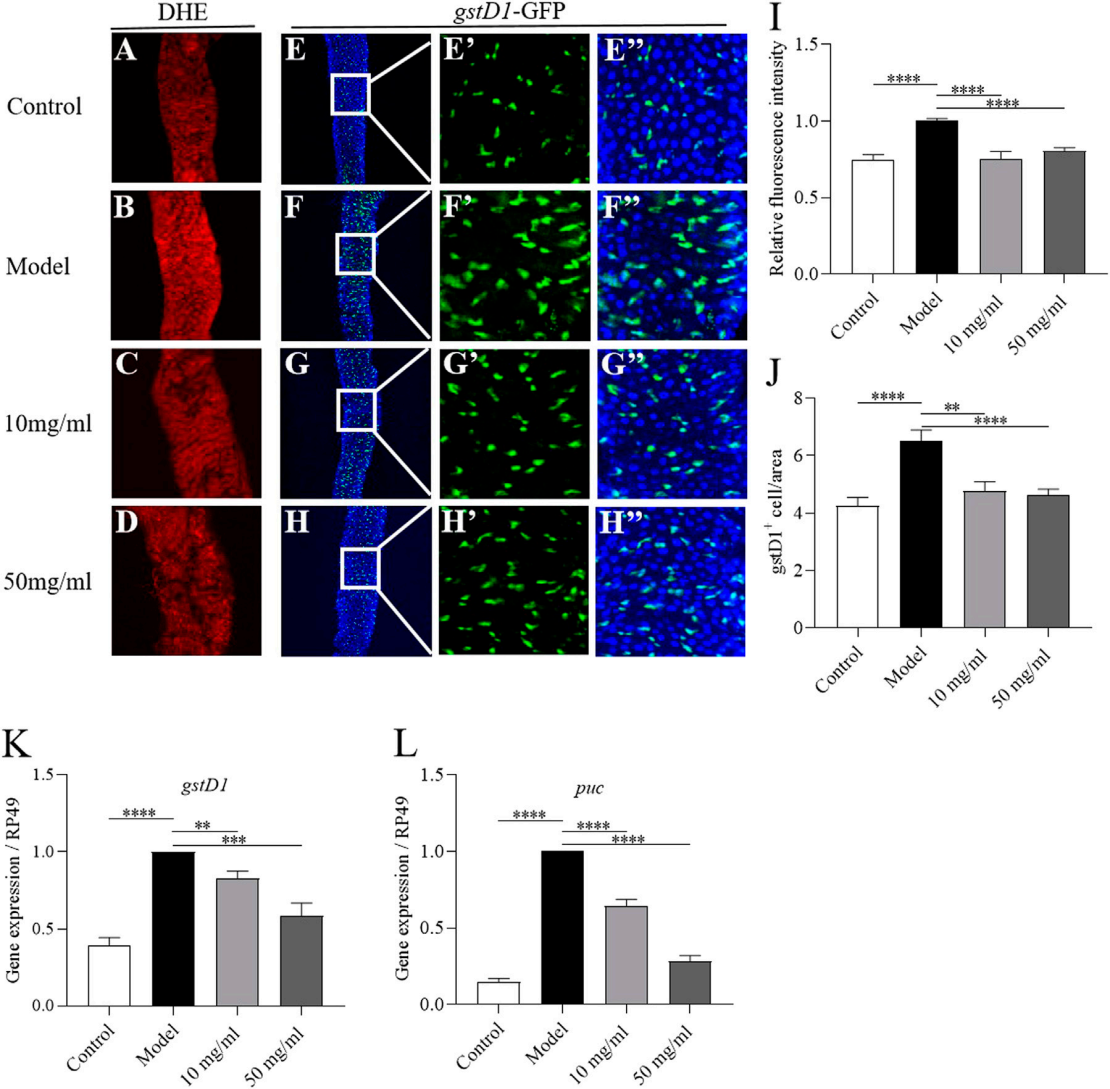
AME alleviates SDS-induced intestine damage by inhibiting oxidative stress-associated JNK signaling. ROS level was monitored by using DHE (red). After treatment with SDS for 72 h, ROS levels were significantly increased in the gut **(A,B)**. AME supplementation reduced the SDS-induced increased ROS levels **(C,D)**. Quantification of the ROS level (I) (*n* = 7–26). Immunofluorescence images of the dissected midguts of gstD1-GFP (green) in flies. After treatment with SDS for 96 h, the GFP levels were remarkably increased **(E,F)**. Supplementation of AME at 10 mg/ml and 50 mg/ml significantly inhibited the expression of gstD1-GFP **(G,H)**. Quantification of the number of gstD1 positive cell **(J)** (*n* = 16–21). 10 mg/ml and 50 mg/ml AME reduced the gene expression of *gstD1* **(K)** and *puc* **(L)** in the gut of SDS-treated flies (*n* = 6–9). The results are presented as the means ± SEM. **p < 0.01, ***p < 0.001, ****p < 0.0001 vs. model group.

Various stresses lead to the production of excessive ROS and damage the host intestinal epithelium (Bhattacharyya et al., 2014). Next we detected whether AME could attenuate the excessive ROS levels in the damaged intestine. ROS level in the intestine was monitored by using dihydroethidium (DHE). After exposed to SDS for 72 h, flies had a robust intensity of fluorescence probe in the posterior midgut compared to control flies (Figures 6A, B, I). 10 mg/ml and 50 mg/ml AME supplementation significantly reversed the SDS-induced ROS accumulation (Figures 6C, D, I). To confirm this finding, we used transgenic flies carrying an independent oxidative stress reporter gene gstD1-GFP (Sykiotis and Bohmann, 2008). The level of GFP was remarkably higher in the intestinal epithelium of SDS-treated flies compared to that of control flies (Figures 6E, F, J), which was consistent with previous study that the activity of gstD1 is increased in differentiated cells and tissues in response to stress (Tan et al., 2018). 10 mg/ml and 50 mg/ml AME supplementation significantly inhibited the gstD1-GFP expression and mRNA level of *gstD1* in SDS-treated flies (Figures 6G, H, J, K). Therefore, these results suggested that AME protected the intestinal epithelium against SDS-induced oxidative damage.

Previous studies have shown that the ROS accumulation enhances the level of JNK signaling, which can promote ISC proliferation (Nagai et al., 2020). Then, we tested whether AME could attenuate JNK pathway in the intestine. The transcriptional level of puc, a reporter of JNK signaling, was significantly increased in the intestine of SDS-treated flies compared to that of control flies (Figure 6L). 10 mg/ml and 50 mg/ml AME supplementation remarkably decreased the expression of puc. Therefore, these results suggested that AME restrained SDS-induced intestinal epithelium *via* suppressing oxidative stress-associated JNK signaling.

### Identification of bioactive ingredients in AME

**FIGURE 7:**
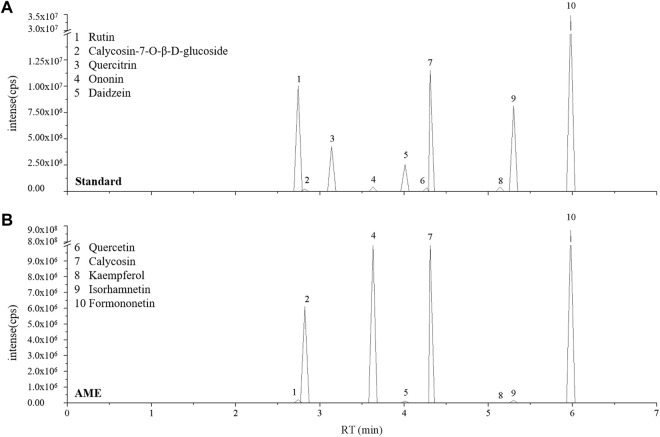
AME flavonoids active ingredient analysis. LC-MS ion-flow chromatogram of Standard **(A)** and AME **(B)**. (1) Rutin, (2) Calycosin-7-O-β-D-glucoside, (3) Quercitrin, (4) Ononin, (5) Daidzein, (6) Quercetin, (7) Calycosin, (8) Kaempferol, (9) Isorhamnetin, (10) Formononetin.

The primary constituent of AM is polysaccharides, saponins, flavonoids, and amino acids. In particular, Calycosin-7-O-β-D-glucoside as one of the flavonoids is used as chemical marker in quality analyses of AM (Song et al., 2007). We identified and quantified some isoflavonoids of flavonoids in AME using LC-MS analysis. The results showed that AME contained calycosin-7-O-β-D-glucoside, rutin, ononin, daidzein, calycosin, kaempferol, isorhamnetin and formononetin (Figure 7), which could be used to screen the functional compound that against SDS-induced inflammatory injury *in vivo*.

### Key compounds of AM alleviate SDS-induced inflammatory injury in flies

**FIGURE 8:**
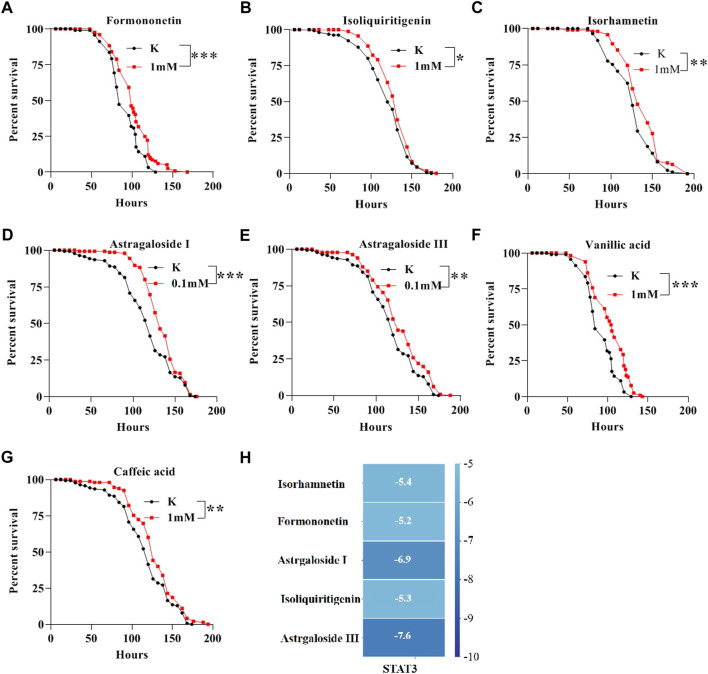
Bioactive compounds of AM alleviate SDS-induced inflammatory injury in flies. The survival curve of flies under SDS-stimulation was extent when flies were fed with 1 mM Formononetin **(A)**, 1 mM Isoliquiritigenin **(B)**, 1 mM Isorhamnetin **(C)**, 0.1 mM Astragaloside I **(D)**, 0.1 mM Astragaloside III **(E)**, 1 mM Vanillic acid **(F)**, 1 mM Caffeic acid **(G)**. **(H)** molecular docking results of the key compounds with STAT3. Log-rank *p* values between survival curves are shown. The results are presented as the means ± SEM. *p < 0.01, **p < 0.01, ***p < 0.001.

To detect the bioactive compounds of AM for treating IBD, the network pharmacology analysis was used. 52 ingredients were screened in AM, which may protect against IBD (Supplementary Table S1). To further exam bioactive ingredients that have anti-inflammatory function *in vivo*, we determined the survival rate of 22 ingredients at 1 mM, Astragaloside I at 0.1 mM, and Astragaloside III at 0.1 mM following SDS treatment (Figure 8 and Supplementary Table S2). The lifespan of flies under SDS stimulation was significantly extented after flies were fed with 7 ingredients that includes formononetin (Figure 8A), isoliquiritigenin (Figure 8B), isorhamnetin (Figure 8C), astragaloside I (Figure 8D), astragaloside III (Figure 8E), vanillic acid (Figure 8F), caffeic acid (Figure 8G), indicating that these compounds have protecive function to against SDS-induced inflammatory injury. To explore whether these compounds target STAT3 protein that is important target for the IBD treatment, we used its crystal structure docked with corresponding molecules. The results showed that docking scores of 5 compounds with STAT3 were below -5 (Kcal/mol). Astragaloside III, astragaloside I, isorhamnetin, isoliquiritigenin and formononetin had a good docking affinity with STAT3 (Figure 8H and Supplementary Figure S2). Thus, these data indicate that formononetin, isoliquiritigenin, isorhamnetin, astragaloside I, astragaloside III, vanillic acid, caffeic acid in AM play an important role in inhibiting inflammatory damage.

## Discussion

**FIGURE 9:**
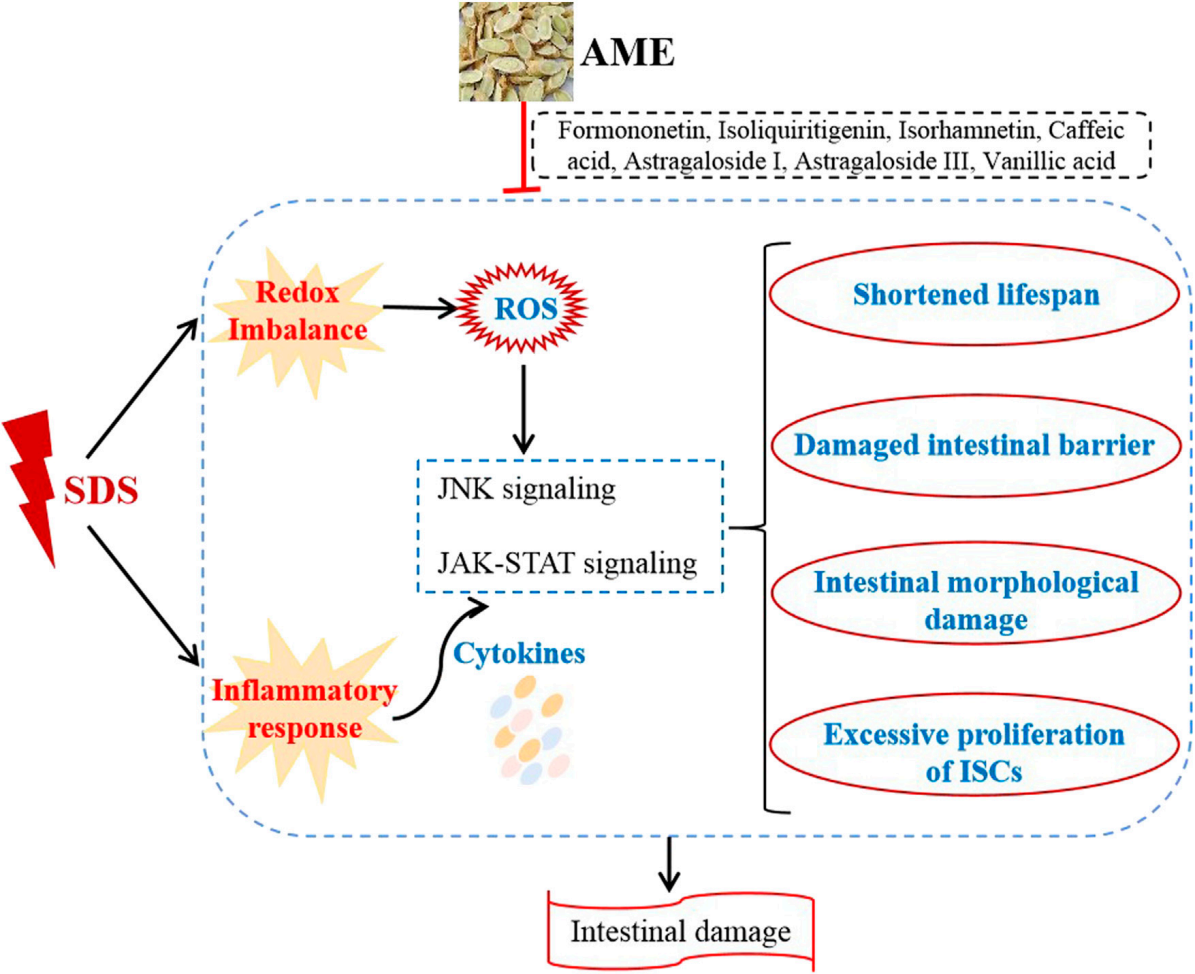
Mechanism of action of AME in the treatment of IBD.

The gastrointestinal tract forms the largest and most important immune epithelial barrier that protects the organism against external dangers (Capo et al., 2019). The intestinal integrity damage is one of the primary causes of various intestinal disorders. AME has exhibited the biological activities, such as antioxidant, anti-inflammatory, and immune activation (Li et al., 2020). In this study, we investigated the role of AME on protecting against SDS-induced intestinal damage. The results showed that oral administration of AME remarkably increases the survival rate, protects against intestinal morphological damage, and inhibits the ISC hyperproliferation in SDS-treated flies, which is mainly regulated by suppressing oxidative stress-associated JNK signaling and JAK-STAT signaling. In addition, formononetin, isoliquiritigenin, isorhamnetin, astragaloside I, astragaloside III, vanillic acid, caffeic acid in AM had the role to inhibit SDS-induced inflammatory damage (Figure 9).

The fruit fly *Drosophila* has been proved as an excelent *in vivo* model for dissecting mechanism and drug screening of various disease, such as cancer, intestinal disease and neurodegenerative disease (Gonzalez, 2013; He et al., 2021; He et al., 2022). Fly midgut is an attractive system to detect the intestinal inflammatory disease, due to its similar anatomical features with mammal intestine and its conserved molecular pathways with mammals (Medina et al., 2022). In this study, we found AME supplementation significantly improve the survival rate following SDS treatment in male and female flies, which means AME has protective function against SDS-induced body injury. AME supplementation could restore the disrupted intestinal barrier integrity, shorted intestinal length, and appeared melanotic tumors in SDS-induced fly IBD model. Consistently, the similar phenotype was observed in rodents, in which AME has been reported to reduce LPS or 2,4-Dinitrobenzene sulfonic acid (DNBS)-induced by intestinal mucosal damage and to inhibit expression of inflammatory cytokine in rats (Ko and Chik, 2009; Cui et al., 2018). Under toxic chemical stimulation, the epithelial cells are damaged in flies, leading to the activation of ISC proliferation to regenerate the damage intestinal epithelium (Buchon et al., 2009; Zhang et al., 2020). Our results shown that AME supplementation protected intestinal epithelial cell damage and restrained abnormal ISC proliferation induced by SDS in flies. These suggest that AME protects against SDS-induced intestine damage. Overall, the protective effects of AME on ulcerative colitis deserve more attention to human health.

ROS accumulation leads to the cellular redox imbalance and serves as critical intracellular messengers in the human body (Amcheslavsky et al., 2009; Wisidagama and Thummel, 2019). Excessive ROS have been implicated in SDS-induced intestinal disorders (Bhattacharyya et al., 2014). Indeed, our results have demonstrated that SDS stimulation leaded to accumulation of ROS in midgut, and oral administration of AME in SDS-treated flies significantly decreased the ROS level, indicating that AME could prevent intestinal disorders *via* inhibiting the ROS accumulation in intestine. This finding corroborates previous reports on the antioxidant effects of AME *in vitro* IECs (Adesso et al., 2018). Elevated ROS enhances the level of JNK signaling and promotes ISC proliferation (Nagai et al., 2020). JAK-STAT signaling is another pathway to restore ISC overproliferation during inflammation (Wei et al., 2022). We found that AME supplementation decreased the SDS-induced increased JNK signaling and JAK-STAT signaling pathways in intestine. Thus, we concluded that AME supplementation inhibited SDS-induced ISC over-proliferation mainly *via* suppressing oxidative stress-associated JNK signaling and JAK-STAT signaling pathways. Previous study have shown that astragaloside IV as one important compound of AM can inhibit neuronal cell apoptosis by inhibiting JNK signaling pathway *in vitro* (You et al., 2019). It is unclear whether there are other pathways that participate in this process. We next employed a network pharmacology approach, combined with molecular docking, to investigate the mechanisms of AME for the treatment of colite ulcerativa (UC). Our network pharmacology analysis show that many targets are enriched in inflammation-related signaling pathways, such as JAK-STAT signaling pathway, NF-kappaB signaling pathway, PI3K-Akt signaling pathway and Toll-like receptor signaling pathway (Supplementary Figure S1). Various studies have shown that AM could suppress inflammatory response and anti-tumor mainly *via* regulating the PI3K-Akt and NF-kappaB signaling pathways (Li et al., 2020). Detailed molecular pathways of AME in treating IBD need to be further explored by using transcriptomics and proteomics in the future, and further experimental validation is needed.

Various isoflavones have been found in AM(Peng et al., 2022). Here, eight active isoflavones were identified as rutin, calycosin-7-O-β-D-glucoside, ononin, daidzein, calycosin, kaempferol, isorhamnetin and formononetin in AME using LC-MS techniques, which is consistent with previous reports (Shan et al., 2019; Peng et al., 2022). According to the active components of AM for treating IBD chose by using network pharmacology analysis (Supplementary Table S1), 24 components were used to detect the anti-inflammatory effect in adult flies. We found that formononetin, isoliquiritigenin, isorhamnetin, astragaloside I, astragaloside III, vanillic acid, caffeic acid in AM have protective function against SDS-induced inflammatory injury. Studies have shown that STAT3 is a key target for the treatment of IBD, so we used STAT3 as target for molecular docking (Lu et al., 2016; Kasembeli et al., 2018) (Supplementary Table S2). Results indicated that Astragaloside III, Astragaloside I, Isorhamnetin, Isoliquiritigenin and Formononetin have good docking affinity with STAT3, indicating that these compounds of AM could directly inhibiting JAK-STAT signaling to treat inflammatory intestinal damage. However, the exactly function and mechanism of these active components against inflammatory intestinal injury are still unclear, which needs to be further elucidated using various IBD models, such as cell, fly and rodents.

In summary, the function and mechanism of AME against inflammatory intestinal injury are evaluated by using chemical toxics-induced gut damage model. As a result, it is shown that AME could increase the survival rate of flies under SDS-feeding condition, protect the intestinal integrity and decrease the enhanced deaths of intestinal epithelial cells. AME protects against SDS-induced intestinal injury mainly through ROS-JNK signaling and JAK-STAT signaling. Further studies showed that formononetin, isoliquiritigenin, isorhamnetin, astragaloside I, astragaloside III, vanillic acid, caffeic acid in AM have protective function against SDS-induced inflammatory injury. Therefore, these results provide evidence for AME to be potentially developed as promising alternative functional food and medicines for the treatment of intestinal diseases; however, the further preclinical studies will be necessary.

## Data Availability

The original contributions presented in the study are included in the article/Supplementary Materials, further inquiries can be directed to the corresponding authors.

## Supplementary material

SUPPLEMENTARY FIGURE S1
**(A)** GO enrichment of the active compounds of AM; **(B)** Astragalus anti-inflammatory target degree ranking top 20 mulberry bubble diagram.

SUPPLEMENTARY FIGURE S2 Analysis of target-compound docking simulation with STAT3. **(A)** Astragaloside III, **(B)** Astragaloside I, **(C)** Isorhamnetin, **(D)** Isoliquiritigenin and **(E)** Formononetin.
