# Supplementary figures and images for: Protective effect of astragalus membranaceus and its bioactive compounds against the intestinal inflammation in Drosophila
Source: Front Pharmacol. 2022 Dec 12;13:1019594. doi: 10.3389/fphar.2022.1019594 (PMC9792096; doi:10.3389/fphar.2022.1019594)

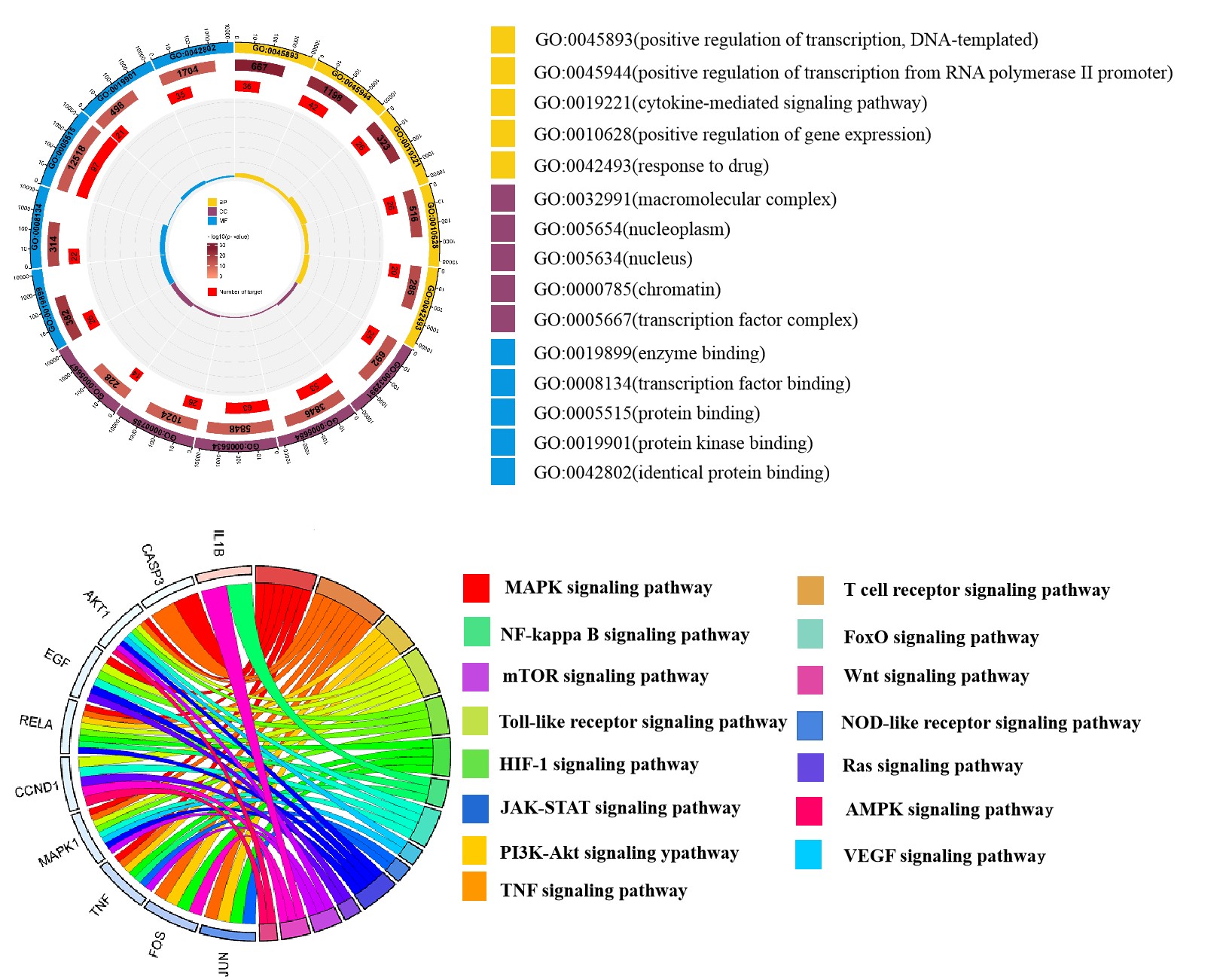

Supplement: Supplementary file 1 [file Image1.JPEG]

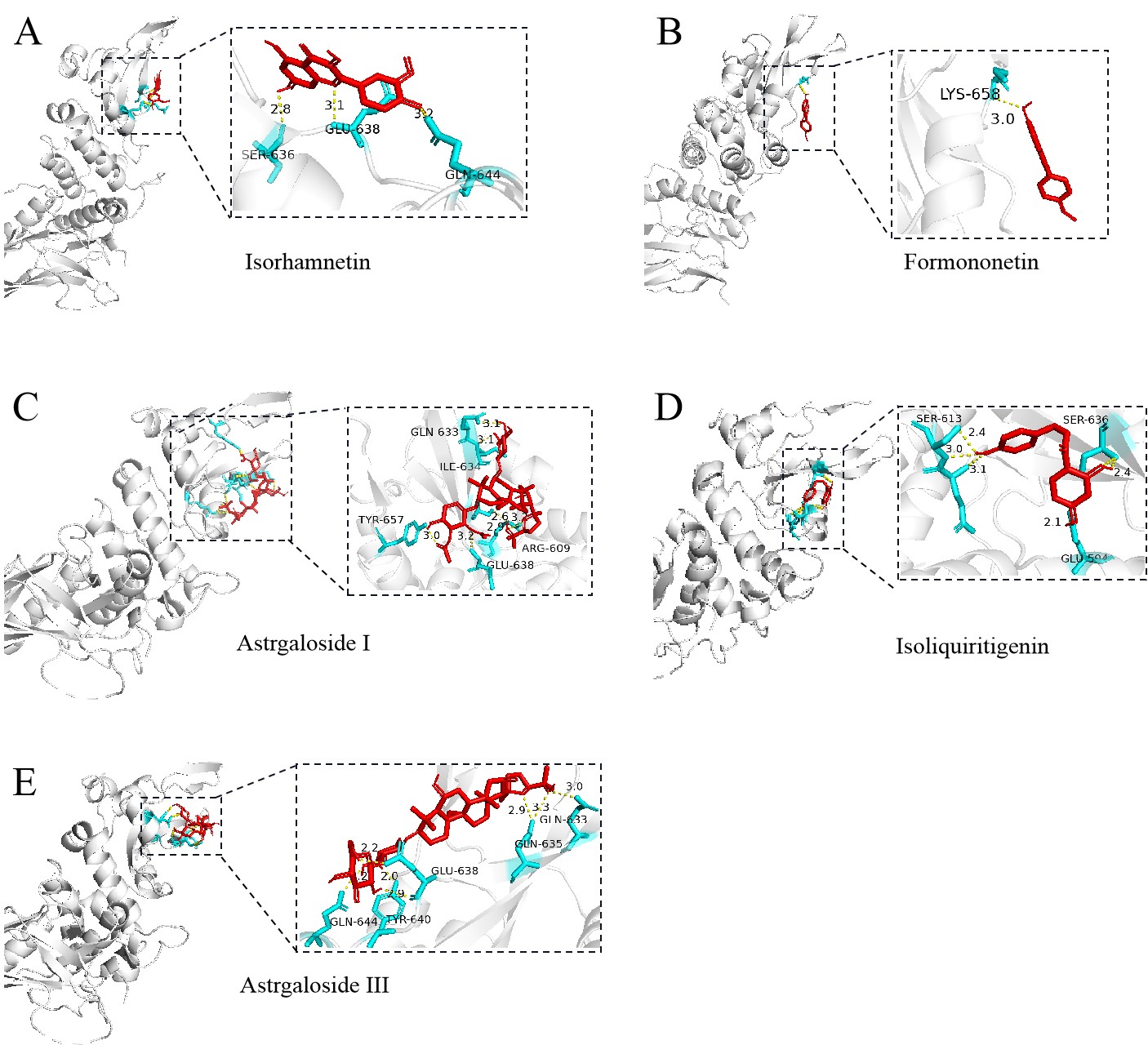

Supplement: Supplementary file 2 [file Image2.JPEG]
