# Supplementary material for: Protective effect of astragalus membranaceus and its bioactive compounds against the intestinal inflammation in Drosophila
Source: Front Pharmacol. 2022 Dec 12;13:1019594. doi: 10.3389/fphar.2022.1019594 (PMC9792096; doi:10.3389/fphar.2022.1019594)
Supplement: Supplementary file 3 [file DataSheet1.docx]

Table S1. **Screening of *Astragalus membranaceus* active ingredients for the treatment of IBD using a network pharmacology approach**

| MOLID | Molecule Name |
| --- | --- |
| MOL000054 | [DL-Arginine](https://www.chemsrc.com/en/cas/7200-25-1_600690.html) |
| MOL000061 | Prolinum |
| MOL000069 | palmitic acid |
| MOL000098 | Quercetin |
| MOL000114 | Vanillic acid |
| MOL000131 | Linoleic Acid |
| MOL000211 | [Betulinic acid](https://www.chemsrc.com/en/cas/472-15-1_894732.html) |
| MOL000239 | Jaranol |
| MOL000251 | Rhamnocitrin |
| MOL000295 | Alexandrin |
| MOL000296 | Hederagenin |
| MOL000354 | Isorhamnetin |
| MOL000356 | Lupeol |
| MOL000371 | 3,9-di-O-methylnissolin |
| MOL000372 | 3-Hydroxy-2-picoline |
| MOL000373 | 5-O-Methylvisammioside |
| MOL000375 | 5'-hydroxyiso-muronulatol-2',5'-di-O-glucoside |
| MOL000376 | 7,2'-dihydroxy-3',4'-dimethoxyisoflavone-7-O-β-D-glucoside |
| MOL000378 | 7-O-methylisomucronulatol |
| MOL000379 | 9-O-Methylnissolin  3-O |
| MOL000380 | [Methylnissolin](https://www.chemsrc.com/en/cas/73340-41-7_666692.html) |
| MOL000381 | Coriolic acid |
| MOL000387 | Bifendate |
| MOL000388 | Gamma-aminobutyric acid |
| MOL000389 | [Ferulic acid](https://www.chemsrc.com/en/cas/1135-24-6_245642.html) |
| MOL000390 | Daidzein |
| MOL000391 | Ononin |
| MOL000392 | Formononetin |
| MOL000396 | (+)-Syringaresinol |
| MOL000397 | [Cis-4-coumaric acid](https://www.chemsrc.com/en/cas/4501-31-9_1035855.html) |
| MOL000400 | Flavaxin |
| MOL000411 | Astraisoflavanin |
| MOL000412 | Mucronulatol |
| MOL000414 | [Caffeic acid](https://www.chemsrc.com/en/cas/331-39-5_1099396.html) |
| MOL000415 | [Rutin](https://old.tcmsp-e.com/molecule.php?qn=415) |
| MOL000416 | Lariciresinol |
| MOL000417 | Calycosin |
| MOL000418 | [Calycosin-7-O-β-D-glucoside](https://www.chemsrc.com/en/cas/20633-67-4_844269.html) |
| MOL000421 | Nicotinic acid |
| MOL000422 | Kaempferol |
| MOL000423 | Rhamnocitrin-3-O-glucoside |
| MOL000424 | [6-Deoxy-L-mannosehydrat](https://www.chemsrc.com/en/cas/10030-85-0_951377.html) |
| MOL000429 | Crystal VI |
| MOL000430 | Betaine |
| MOL000431 | Coumarin |
| MOL000432 | Linolenic acid |
| MOL000433 | [Folic Acid](https://www.chemsrc.com/en/cas/59-30-3_1191873.html) |
| MOL000436 | [Isoliquiritigenin](https://www.chemsrc.com/en/cas/961-29-5_894793.html) |
| MOL000437 | [Isoquercitrin](https://www.chemsrc.com/en/cas/21637-25-2_599763.html) |
| MOL000439 | Isomucronulatol-7,2'-di-O-glucosiole |
| MOL000442 | 1,7-Dihydroxy-3,9-dimethoxy pterocarpene |
| MOL005928 | [Isoferulic acid](https://old.tcmsp-e.com/molecule.php?qn=5928) |

Table S2. **The active ingredients of *Astragalus membranaceus* with anti-inflammatory activity**

| Molecule Name | Mean ± SD lifespan (day) | | *p* |
| --- | --- | --- | --- |
|  | Control | Drug |  |
| [Quercetin](https://www.chemsrc.com/en/cas/117-39-5_947030.html) | 109.76±21.81 | 116.18±24.19 | 0.64 |
| [Isoquercitrin](https://www.chemsrc.com/en/cas/21637-25-2_599763.html) | 121.5±31.10 | 108.59±24.91 | 0.45 |
| Kaempferol | 108.67±10.25 | 106.69±3.60 | 0.66 |
| Daidzein | 117.69±17.06 | 115.91±18.67 | 0.86 |
| [Calycosin](https://www.chemsrc.com/en/cas/20575-57-9_99847.html) | 108.59±27.65 | 102.10±26.14 | 0.69 |
| Calycosin-7-O-β-D-glucoside | 105.25±8.17 | 101.76±8.22 | 0.41 |
| [Rutin](https://www.chemsrc.com/en/cas/153-18-4_894794.html) | 156.39±5.98 | 156.47±7.89 | 0.98 |
| [Ononin](https://www.chemsrc.com/en/cas/486-62-4_1106139.html) | 105.25±8.17 | 100.6±9.25 | 0.31 |
| [Betulinic acid](https://www.chemsrc.com/en/cas/472-15-1_894732.html) | 119.55±17.71 | 116.60±9.54 | 0.73 |
| [Astragaloside II](https://www.chemsrc.com/en/cas/84676-89-1_402120.html) | 126.01±13.42 | 120.01±8.23 | 0.37 |
| [Astragaloside Ⅳ](https://www.chemsrc.com/en/cas/84676-89-1_402120.html) | 137.03±34.60 | 133.67±46.49 | 0.94 |
| [beta-Sitosterol](https://www.chemsrc.com/en/cas/83-46-5_749358.html) | 109.76±21.81 | 110.87±16.83 | 0.92 |
| [Lupeol](https://www.chemsrc.com/en/cas/545-47-1_315301.html) | 126.35±6.45 | 125.71±9.09 | 0.89 |
| Heriguard | 112.06±12.86 | 97.61±15.79 | 0.063 |
| [Isoferulic acid](https://www.chemsrc.com/en/cas/25522-33-2_601405.html) | 114.84±36.17 | 139.73±13.85 | 0.15 |
| [Cis-4-coumaric acid](https://www.chemsrc.com/en/cas/4501-31-9_1035855.html) | 119.55±17.71 | 119.43±2.88 | 0.99 |
| [Ferulic acid](https://www.chemsrc.com/en/cas/1135-24-6_245642.html) | 126.61±13.42 | 127.66±12.05 | 0.83 |
